# ﻿*Carexrecondita* Muñoz-Schüler, Martín-Bravo & Jim.Mejías (Carex section *Junciformes* Kük., Cyperaceae), a new sedge species from the Andes of central Chile

**DOI:** 10.3897/phytokeys.243.115991

**Published:** 2024-06-19

**Authors:** Paulo Muñoz-Schüler, Ana Morales-Alonso, José Ignacio Márquez-Corro, Mary T. K. Arroyo, Santiago Martín-Bravo, Pedro Jiménez-Mejías

**Affiliations:** 1 Herbario CONC, Departamento de Botánica, Facultad de Ciencias Naturales y Oceanográficas, Universidad de Concepción, Casilla 160-C, Concepción, Chile Universidad de Concepción Concepción Chile; 2 Area de Biodiversidad y Conservación, Departamento de Biología, Física y Química Inorgánica, Universidad Rey Juan Carlos, Madrid, Spain Universidad Rey Juan Carlos Madrid Spain; 3 Área de Botánica, Departamento de Biología Molecular e Ingeniería Bioquímica, Universidad Pablo de Olavide, Carretera de Utrera km 1 sn, 41013 Sevilla, Spain Universidad Pablo de Olavide Sevilla Spain; 4 Jodrell Laboratory, Royal Botanic Gardens, Kew, TW9 3AB, Richmond, London, UK Royal Botanic Gardens, Kew London United Kingdom; 5 Departamento de Ciencias Ecológicas, Universidad de Chile, Las Palmeras 3425, 7800003 Ñuñoa, Santiago, Chile Universidad de Chile Santiago Chile; 6 Instituto de Ecología y Biodiversidad (IEB), Las Palmeras 3425, 7800003 Ñuñoa, Santiago, Chile Instituto de Ecología y Biodiversidad (IEB) Santiago Chile; 7 Cape Horn International Center (CHIC), O’Higgins 310, 6350001 Cabo de Hornos, Chile Cape Horn International Center (CHIC) Cabo de Hornos Chile

**Keywords:** Andes, *Carex*, Chile, Cyperaceae, South America, taxonomy

## Abstract

CarexsectionJunciformes is one of the most diverse groups of the genus in South America, consisting of approximately 30 species. Here we describe a new species, *Carexrecondita*, belonging to this section. We studied its placement within a molecular phylogeny of the group and found it to constitute an independent lineage. The new species is morphologically very close to *C.austroamericana*, from southern Patagonia, despite being phylogenetically divergent to the rest of Patagonian species of sect. Junciformes. So far, this species is known only from a few specimens recently collected in its type locality, despite growing in a well-collected area in the Andes of Metropolitana Region of Santiago, the most populated administrative region of Chile. We provide a detailed morphological description, comments on its relationship with other Southern Cone species of sect. Junciformes and relevant ecological notes.

## ﻿Introduction

With about 96 species, *Carex* is among the three most biodiverse genera of the flora of Chile (Rodriguez et al. 2018; Muñoz-Schüler et al. 2023). However, the taxonomic knowledge of the genus in the country is fragmentary, as it is in the rest of South America. Recently, a series of works focusing on particular groups of *Carex* are shedding light on the taxonomic structure and systematic relationships of these understudied groups (e.g. Benítez-Benítez et al. 2021; Jiménez-Mejías et al. 2021a; García‐Moro et al. 2022). The relatively large number of new species that are being steadily described from the continent in the last few years (e.g., Jiménez-Mejías and Escudero 2016; Jiménez-Mejías and Roalson 2016; Poindexter et al. 2017; Jiménez-Mejías and Reznicek 2018; Jiménez-Mejías et al. 2020, 2021a, 2021b) depicts how much is still to be done before a comprehensive understanding of the genus in South America is reached.

CarexsectionJunciformes Kük (subgenusPsyllophorae), with over 25 species in the continent (Roalson et al. 2021; Morales-Alonso et al. in press), is one of the most diversified *Carex* groups of South America. It is almost entirely endemic to the continent, with only four species in the SW Pacific and a clear center of diversity in Patagonia (Barros 1935, 1969; Moore 1968, 1983). The evolutionary history of the group has been recently studied by Benítez-Benítez et al. (2021). They showed that the section includes two main lineages, namely “Junciformes clade” and “Aciculares clade” -as these were formerly considered independent sections due to several diagnostic morphological characters (Wheeler 1989)- plus three isolated lineages (*C.camptoglochin* V.I.Krecz., *C.phalaroides* Kunth s.l., and *C.vallis-pulchrae* Phil.). Benítez-Benítez et al. (2021) also showed that the ancestral area and diversification cradle of the group was centered in South America, with several migrations out of the Southern Cone to the Tropical Andes, Brazil, and the SW Pacific. However, the circumscription of this section is still under study, as a recent phylogenetic framework excluded the geographically isolated Pampean *C.herteri* G.A.Wheeler from sect. Junciformes (Morales-Alonso et al. in press).

During a fieldtrip in Chile in January 2023 we collected material of an undetermined acaulescent Carexsect.Junciformes taxon in the Andes of Metropolitana region of Santiago (see Taxonomic Treatment for details on the collection). In a preliminary assessment using the available identification keys (Barros 1969; Moore 1983; Wheeler 1986, 1988) the specimen was determined as *C.austroamericana* G.A. Wheeler, a Patagonian species belonging to the Junciformes clade (Benítez-Benítez et al. 2021). The strong disjunction of this undetermined specimen population regarding its tentative species distribution (>700 km north) raised the question of whether it could be a different species. Accordingly, we proceeded with a molecular phylogenetic and morphological study to resolve the taxonomic identity and systematic affinities of the problematic specimen, and to warrant its taxonomic recognition if required.

## ﻿Materials and methods

### ﻿Molecular analyses

Silica-dried leaves of the undetermined taxon collected on the field in January 2023 were used for DNA extraction and PCR amplification.

The DNA extraction was performed using a modified CTAB procedure (Doyle and Doyle 1987). We amplified the loci ETS and ITS (nuclear ribosomal DNA, nrDNA) and *mat*K and *rps*16 (plastidial DNA, ptDNA) since these have been previously used for the study of the systematics of sect. Junciformes, following the protocols in Benítez-Benítez et al. (2021). The amplified products were externally sequenced by Macrogen (Madrid, Spain). The sequence chromatograms were edited and revised with Geneious v.2022.0.1 (Biomatters Ltd., Auckland, New Zealand). The resulting sequences were concatenated and included in the complete multiaccession matrix built by Benítez-Benítez et al. (2021). The alignment was made with MUSCLE v.3.8.425 (Edgar 2004), and the best substitution models for each DNA region were selected with JModelTest 2 (Guindon and Gascuel 2003; Darriba et al. 2012) as implemented in CIPRES Science Gateway v3.3 (www.phylo.org/portal2; Miller et al. 2010). The concatenated matrix contained 106 accessions, 96 belonging to species of subg. Psyllophorae of which 17 are representatives of the South American species of sect. Junciformes. Thus, all but five species -if *C.phalaroides* is considered in a broad sense (Morales-Alonso and Jiménez-Mejías 2021)- from the section were included in the study. The missing species are *C.boelckeiana* Barros, *C.moorei* G.A.Wheeler, and *C.nelmesiana* Barros, which are morphologically close to the Junciformes s.s. clade, and *C.sanctae-marthae* L.E.Mora & J.O.Rangel and *C.transandina* G.A.Wheeler, putatively belonging to the Aciculares clade. It is worth saying that the samples determined as *C.nelmesiana* and *C.transandina* in Benítez-Benítez et al. (2021) were re-identified here as *C.austroamericana* (P.J.-M., pers. obs.). Additionally, the former species named *C.lateriflora* Phil. has been here corrected to *C.trichodes* Steudel ex Boott (Penneckamp 2022).

The phylogenetic reconstructions analysis were run employing Maximum Likelihood (ML) in RAxML v.8.2.12 (Stamatakis 2014), and Bayesian Inference (BI) using MrBayes v.3.2 (Ronquist and Huelsenbeck 2003; Ronquist et al. 2012) as implemented in the CIPRES Science Gateway v3.3 (Miller et al. 2010) and in the Scientific Computer Center of Andalucía supercomputing cluster (CICA). ML analysis was set with default parameters and 1000 replicates. BI analysis was set with 10 million generations and 4 simultaneous Monte Carlo Markov chains (MCMC) runs, with a sample frequency of one tree every 1000 generations. After checking for the analysis to reach stationarity, a burn-in threshold of 25% was applied and the consensus tree was built using the 50% majority rule. Strong clade support was considered above 75% for bootstrap (BS) and above 0.90 for posterior probability (PP) (Gehrke et al. 2010).

### ﻿Morphological study

Three collections of the undetermined taxon were located and studied: the original collection from the 2023 fieldwork campaign, a second collection by M.T.K.A. in 2022, and a last voucher located in CONC collection, determined as *Carexandina* Phil., and collected in 2007. These vouchers were determined using the available keys and thoroughly compared against descriptions (Barros 1969; Moore 1983; Wheeler 1986, 1988; see Results).

Direct comparisons were also carried out against material (including type material -herbarium specimens or high-resolution images of them-) belonging to the other three Junciformes clade acaulescent species: *C.argentina* Barros, *C.austroamericana*, and *C.nelmesiana*. In total, we studied 17 vouchers housed at BAB, CONC, SI, and UPOS.

### ﻿Conservation status

Since our results showed that the undetermined taxon constitutes an undescribed species with a very restricted known distribution, a preliminary conservation status for it was assessed. We followed the criteria provided in the IUCN Red List Categories and Criteria Version 13 (IUCN 2017) manual.

## ﻿Results

### ﻿Molecular analyses

The multiaccession matrix consisted of 606 bp for ETS, 624 bp for ITS, 572 bp for *mat*K, and 937 bp for *rps*16. The length of obtained sequences for the problematic specimen were 493 bp for ETS, 603 bp for ITS, 503 bp for *matK*, while for *rps*16 marker only 285 bp could be obtained. The obtained BI tree topology (Fig. 3) was highly in concordance with the one recovered in Benítez-Benítez et al. (2021) and Morales-Alonso et al. (unpublished results). The best resolved topology was obtained with BI with no indels coded. ML and BI analyses resulted in very similar topologies. The main incongruences were mostly minor and retrieved at shallow branch levels of the tree.

The sample of the undetermined taxon (Fig. 3) was nested within the Junciformes clade with maximum BS and PP support. It was placed as sister to a well-supported clade containing the caulescent *C.sorianoi* Barros and the acaulescent *C.austroamericana* (BS = 100; PP = 1,00). The sample shared up to 98.6% pairwise nucleotide identity with *C.austroamericana* and *C.sorianoi* while 96.5% of identical sites were reached for this clade.

### ﻿Morphological analyses

The samples of the undetermined specimen share a number of morphological affinities with *C.austroamericana*, such as an acaulescent growing habit, stiff leaves that surpass the entire inflorescence, and stipitate utricles. However, it can be distinguished from this and any other species of sect. Junciformes by having the following combination of characters: leaves up to 9 cm long, spikes ≤ 3.5 mm wide, and utricles ellipsoid, ovoid or sub-obovoid, glabrous to sparsely hispidulous, with -usually weakly- raised nerves on the faces and a stipitate base often flattened. A detailed morphological comparison between these problematic specimens, *C.austroamericana* and the other three acaulescent species of sect. Junciformes from the Southern Cone is provided in Table 1.

**Table 1.** Variation in diagnostic morphological characters of *C.recondita* and morphologically related Patagonian species of sect. Junciformes. Characters listed for *C.austroamericana* and *C.nelmesiana* are based largely on Wheeler (1986) and complemented with personal observations and measurements. Given the low sampling sizes, the natural variation of these characters might be underestimated and could be wider.

Character	*Carexaustroamericana*	*Carexnelmesiana*	*Carexargentina*	*Carexrecondita*
Leaf length	0.5–5 cm	1–5 cm	8–12 cm	2.9–9 cm
Spike width	3.5–6 mm	5–6 mm	5 mm	2.8–3.5 mm
Number of female flowers/utricles	Four to six	Four to seven	Five to seven	Four to five
Utricle shape	Obovoid	Sub-globose	Ovate to lanceolate	Elliptical, sub-fusiform or sub-obovate
Utricle indurnenturn	Glabrous, but scaberulent on the beak and the margins of the distal third of the body	Puberulent, margins hispidulous	Flocculent	Glabrous to hispidulous, the margins scaberulent on the distal half of the body or beyond
Utricle venation	Faintly or prominently veined, up to 6–8 prominent veins abaxially and 4–6 veined adaxially	Faintly veined or with 4–7 prominent veins in the proximal third of the abaxial side	Veinless	Weakly 2–5 veined adaxially and 1–4 veined abaxially
Utricle base	Stipitate, rounded or flattened in cross section	Shortly stipitate	Truncate to stipitate	Stipitate and flattened in cross section
Utricle margins	Rounded	Rounded	Rounded	Flattened
Beak	Conical, bidentate	Shortly conical and obscurely bidentate	Longly conical and strongly bidentate	Conical, short and bidentate
Rachilla	Subulate	Ovate	Lanceolate	Linear

From the other three Junciformes clade species not included in the phylogeny (*C.boelckeiana*, *C.moorei* and *C.nelmesiana*) the undetermined taxon is readily distinguished because the two first species have well-developed stems, longer than leaves, as well as larger utricles (see Barros 1969, Wheeler 1988), and *C.nelmesiana* has utricles with faces entirely nerveless. The undetermined taxon is also readily distinguished from the closely related *C.sorianoi* (Fig. 3) because this latter has culms exceeding its leaves, that are usually flexuose, and also by having two stigmatic female flowers (instead of three; see the Description section below).

## ﻿Discussion

The set of evidence presented here makes it clear that the studied specimens should be treated as a new species. Even though it is morphologically very similar to *C.austroamericana*, the molecular data by itself is compelling enough to support its status as a different taxon (Fig. 2). Moreover, the differences in key -but subtle- traits (such as rachilla shape and utricle venation; see Table 1) and its disjunct distribution with respect to the other three phylogenetically related species of southern South America (i.e., *C.austroamericana*, *C.nelmesiana* and *C.sorianoi*) reinforces treating the studied specimens as a new species. Morphologically similar species, such as the species here described, are prevalent within sect. Junciformes despite many of its lineages having deep times of divergence (dating back to Miocene; Benítez-Benítez et al. 2021). The relatively reduced diversification rate estimated for subgen. Psyllophorae suggests that its diversity stems from the progressive accumulation of morphological changes (disparification) and thus might have resulted in lower levels of morphological diversity than the expected by means of evolutionary radiations (Benítez-Benítez et al. 2021). This overall morphological resemblance has led to the misuse of names in quite a few cases for Patagonian taxa (Barros 1969, Wheeler and Muñoz-Schick 1990), a recurrent phenomenon in *Carex* that hinders its already problematic taxonomy (Jiménez‐Mejías et al. 2014). Likewise, Wheeler (1986) highlighted the resemblance of *C.austroamericana* with *C.argentina* and *C.nelmesiana*, but the morphological differences between those three species were clearer than that existing between the new species and *C.austroamericana* (Table 1).

Wheeler (1986) described the phytogeography of *C.austroamericana* as growing primarily on Patagonian steppe, forming “*small, dense tufts in moist depressions of the steppe, which is often dominated by Festuca gracillima*”. For *C.nelmesiana* he said that it “*grows on Patagonian steppe and occur primarily on moderate to steep slopes, where they form dense tufts in dry, exposed sites*”. Currently, both species are known only from stepparian environments in southern Patagonia, ranging from Río Negro to Tierra del Fuego provinces (Argentina), and Magallanes region (Chile) for *C.austroamericana*, and Chubut to Santa Cruz provinces (Argentina), and Aysén region (Chile) for *C.nelmesiana* (Fig. 4). Another tuft-forming acaulescent species, *C.argentina*, also grows in the Patagonian steppe, but extends its distribution further north through the Andes, reaching the north of Mendoza province (Argentina) and Metropolitana region of Santiago (Chile), and being reported as south as Santa Cruz province (Argentina) and Araucanía region (Chile). The species here described, however, is only known from the Andes of Metropolitana region of Santiago, where it grows in a lower altitudinal vegetation belt than *C.argentina* (2750–2880 vs 3200 m; Fig. 4), and at least 1300 km north of the northernmost population of any of the two other Patagonian species (Fig. 4).

Andean-Patagonian disjunctions are not uncommon within and between closely related species of *Carex* (e.g. South American sect. Racemosae, Wheeler 1990, Jiménez-Mejías et al. 2021b; *C.melanocystis* É.Desv., Villaverde et al. 2017, Jiménez-Mejías et al. 2021a; Muñoz-Schüler et al. 2023; *C.microglochin* Wahlenb., Wheeler and Guaglianone 2003) and sect. Junciformes is not an exception (e.g. *C.camptoglochin*, *C.phalaroides*, C.vallis-pulchraevar.vallis-pulchrae; Wheeler and Guaglianone 2003; Wheeler 1989; Benítez-Benítez et al. 2021). This apparent gap might be either due to the lack of suitable environments in the territory between the two groups of known populations, or a reflection of the undersampling in Andean steppe-like environments in south-central Southern Cone in general, and of *Carex* species in particular.

## ﻿Taxonomic treatment

### ﻿ Carex recondita

Taxon classificationPlantaePoalesCyperaceae

Muñoz-Schüler, Martín-Bravo & Jim.Mejías sp. nov.

090BCA17-4F2A-5E80-82FD-3CEBA1C2D468

urn:lsid:ipni.org:names:77343736-1

#### Diagnosis.

*Carexrecondita* is similar to *C.austroamericana*, from which it is distinguished by its longer leaves up to 9 cm long (versus 5 cm long in *C.austroamericana*), narrower spikes up to 3.5 mm wide (versus 3.5–6 mm wide), and weakly veined ellipsoid, obovoid or suborbicular utricles, with a flattened stipitate base (versus strongly veined obovoid utricles, with a rounded or flattened stipitate base).

#### Type.

Chile. Región Metropolitana de Santiago: Provincia de Santiago, Farellones, camino al centro de ski Valle Nevado, Tres Puntas, 2750 m.a.s.l., 33°21.39183'S, 70°15.45538'W, 16 January 2023, *P. Jiménez Mejías, J.I. Márquez Corro, S. Martín Bravo & P. Muñoz Schüler 12PJM-CL23* (holotype: CONC 193519; isotypes: EIF 17307, UPOS).

#### Description.

Plants low-growing, densely caespitose 3–10.2 cm tall from the base of the shoots to the tip of the leaves, acaulescent or nearly so. Leaves numerous, up to eight per shoot, much longer than the flowering shoots and concealing them, 2–8.2 cm long, canaliculate but flattish distally, stiff to slightly flexuose. Flowering shoots elevated on erect stalks of 1.2–4.4 cm long formed by the basal leaf sheaths, with the portion between the insertion of the distal-most leaf and the proximal-most bract inconspicuous, up to 1.2 mm long. Inflorescence a single, terminal, androgynous, subglobose spike, 4.3–6.2 mm long and 2.8–3.5 mm wide, subtended by an involucral bract, with a glumaceous base and prolonged into a 5.8–9.1 mm long antrorsely scabrid or smooth setaceous portion. Staminate part concealed by the pistillate portion, 2-flowered. Pistillate part 4–5 flowered, sometimes with an extra infertile flower borne by the involucral bract; glumes ovate to widely elliptical, 3.1–4.1 mm long and 1.4–2.3 mm wide, glabrous, with wide hyaline margins contrasting with a narrow green middle strip, veinless, attenuated distally into an awn, the 2-proximalmost glumes with a 1.9–3.3 mm long awned portion that usually surpasses the whole inflorescence, distalmost glumes with a 0.6–0.9 mm long mucronate apex. Utricles 2.4–3 mm long and 1.2–1.8 mm wide, ellipsoid, suborbicular, ovoid or sub-obovoid, obscurely trigonous in cross section, green to pale green, with 2 prominent lateral veins dark greenish, contrasting with the rest of the body, veinless or 2–5 weakly veined adaxially and 1–4 veined abaxially, glabrous to sparsely hispidulous on its distal half, with the lateral veins hispidulous to ciliolate on its distal ⅔ portion, attenuated or constricted proximally into a sub-stipitate base 0.4–0.8 mm long, often flattened in cross section, constricted distally into a short, bidentate, and pale beak 0.3–0.6 mm long. Achenes 1.9–2.2 long and 1.2–1.6 mm wide, broadly elliptical to sub-obovoid, obscurely trigonous, often flattened, greenish, more or less constricted proximally into a sub-stipitate base and attenuated distally into a short beak. Rachilla often absent, when present 0.6–0.9 mm long, linear, margins smooth. Stigmas 3. Anthers 3.

#### Phenology.

The phenology of this species is poorly understood. A fully flowering specimen with some ripe utricles was collected in mid-late December, while a specimen with entirely ripe utricles bearing some female flowers was collected in mid-January. According to these observations we infer that the flowering period for the new species is likely to range from mid-November to late December with mature individuals bearing ripe utricles from mid-late December onwards. This agrees with the plant community peak flowering period at 2935 m.a.s.l. (late-December to early-January) in an adjacent high Andean valley (Arroyo et al. 1981), thus our species should flower during the first half of the flowering season.

#### Etymology.

The specific epithet *recondita* (female) refers to the Latin word *recondito* (male), meaning something hidden or occult, apropos the acaulescent inflorescence and low-growing habit of this species.

#### Distribution and habitat.

**Figure 1.**
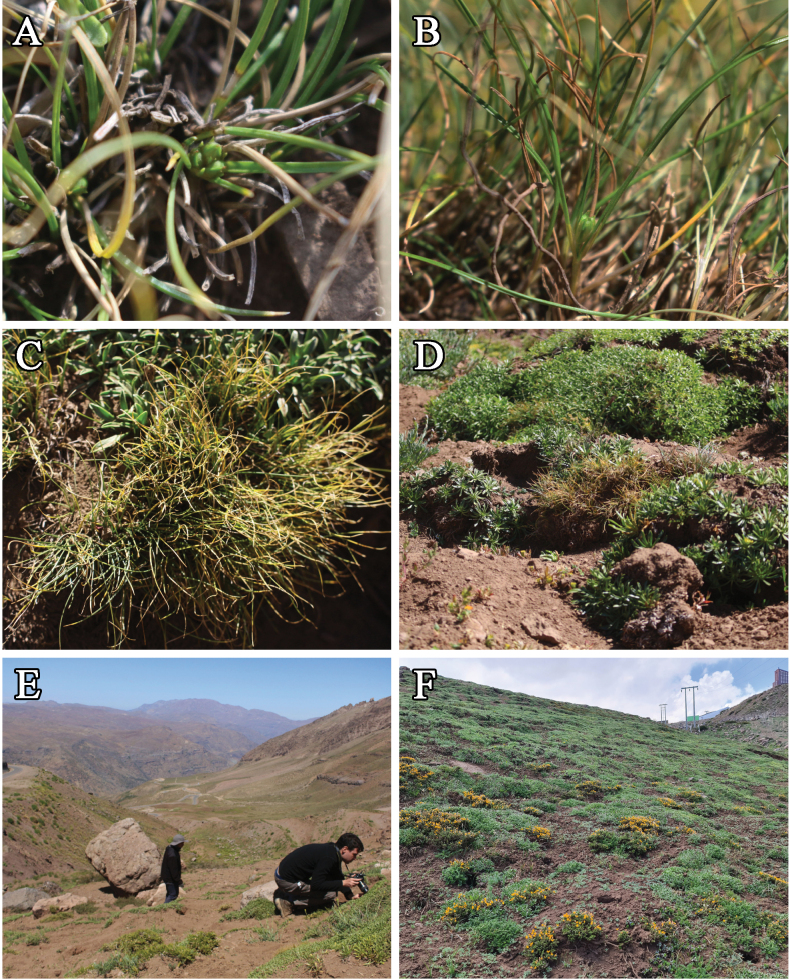
*Carexrecondita* Muñoz-Schüler, Martín-Bravo & Jim.Mejías **A, B** aerial and frontal view of the plant and a spike **C, D** habit **E** two of the authors looking for more specimens of *C.recondita* on its habitat, the road can be seen at the upper-left portion of the picture **F** habitat of *C.recondita*, showing dominant *Azorellaruizii* (green cushions) and *Anarthrophyllumcumingii* (prostrate shrub with orange-yellow flowers). Photos by P M-S (**A–E**) and MTKA (**F**).

*Carexrecondita* occurs in the area of La Parva and Farellones-Valle Nevado ski circuits, in the Andes of the Metropolitana region of Santiago (see Fig. 4), and has been reported to grow between 2750–2880 m.a.s.l. The new species occurs in the central Chilean Andes, in an area characterized by a semi-arid Mediterranean-type influence climate (Arroyo et al. 1981) and it grows in the alpine cushion plant belt (Cavieres et al. 2000), on steep slopes around 650 m of elevation above a climatically depressed *Kageneckiaangustifolia* D.Don treeline. Phytogeographically, this area and elevation in the high Andes falls in the Cuyano-Pikumche district of the Altoandean Province of the southern Andes (Biganzoli et al. 2022). Snow is received from May-June to October-November, depending on the year. *Carexrecondita* was growing on a south-facing slope next to an important road leading from Farellones mountain town to Valle Nevado ski resort, forming dense tufts in a habitat dominated by the cushion-forming umbellifer *Azorellaruizii* G.M. Plunkett & A.N. Nicolas (Fig. 1). Other common species in the area include *Acaenapinnatifida* Ruiz & Pav., *Adesmiacorymbosa* Clos, *Anarthrophyllumcumingii* (Hook. & Arn.) F. Phil., *Chaetantheraeuphrasioides* (DC.) F. Meigen, *Haplopappusscrobiculatus* (Nees) DC., *Hypochaerisclarionoides* (J. Remy) Reiche, *Hordeumcomosum* J. Presl, *Noccaeamagellanica* (Comm. ex Poir.) Holub, *Pereziacarthamoides* (D. Don) Hook. & Arn., *Poaholciformis* J. Presl, *Quinchamaliumchilense* Molina, *Rytidospermapictum* (Nees & Meyen) Nicora and *Seneciopentaphyllus* Phil. The exotic *Taraxacumofficinale* F.H. Wigg., an invasive Palearctic species at high elevations in the central Chilean Andes (Cavieres et al. 2008), can also be locally common. This plant is currently known from only three points. Two are located on two contiguous slopes, with approximately 280 meters of distance between each other, while the third point is located approximately 3 km Northwest in a straight line from the closest known point (Fig. 4). Further fieldwork might expand its known presence within the area by searching in environmentally similar localities.

**Figure 2.**
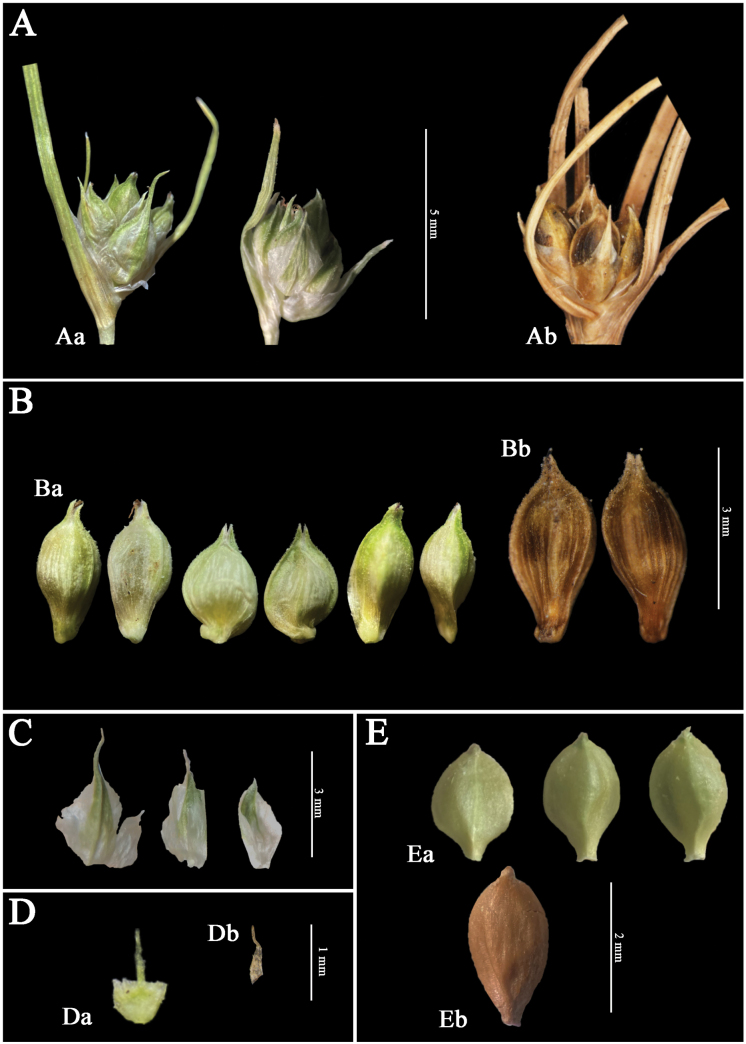
Detailed comparison between *C.recondita* and *C.austroamericana***A** spikes of *C.recondita* (Aa) and *C.austroamericana* (Ab) **B** utricles of *C.recondita* (Ba) and *C.austroamericana* (Bb) **C** glumes of *C.recondita***D** rachillas of *C.recondita* (Da) and *C.austroamericana* (Db). Note that the rachilla of *C.recondita* is attached to the utricle base **E** achenes of *C.recondita* (Ea) and *C.austroamericana* (Eb).

**Figure 3.**
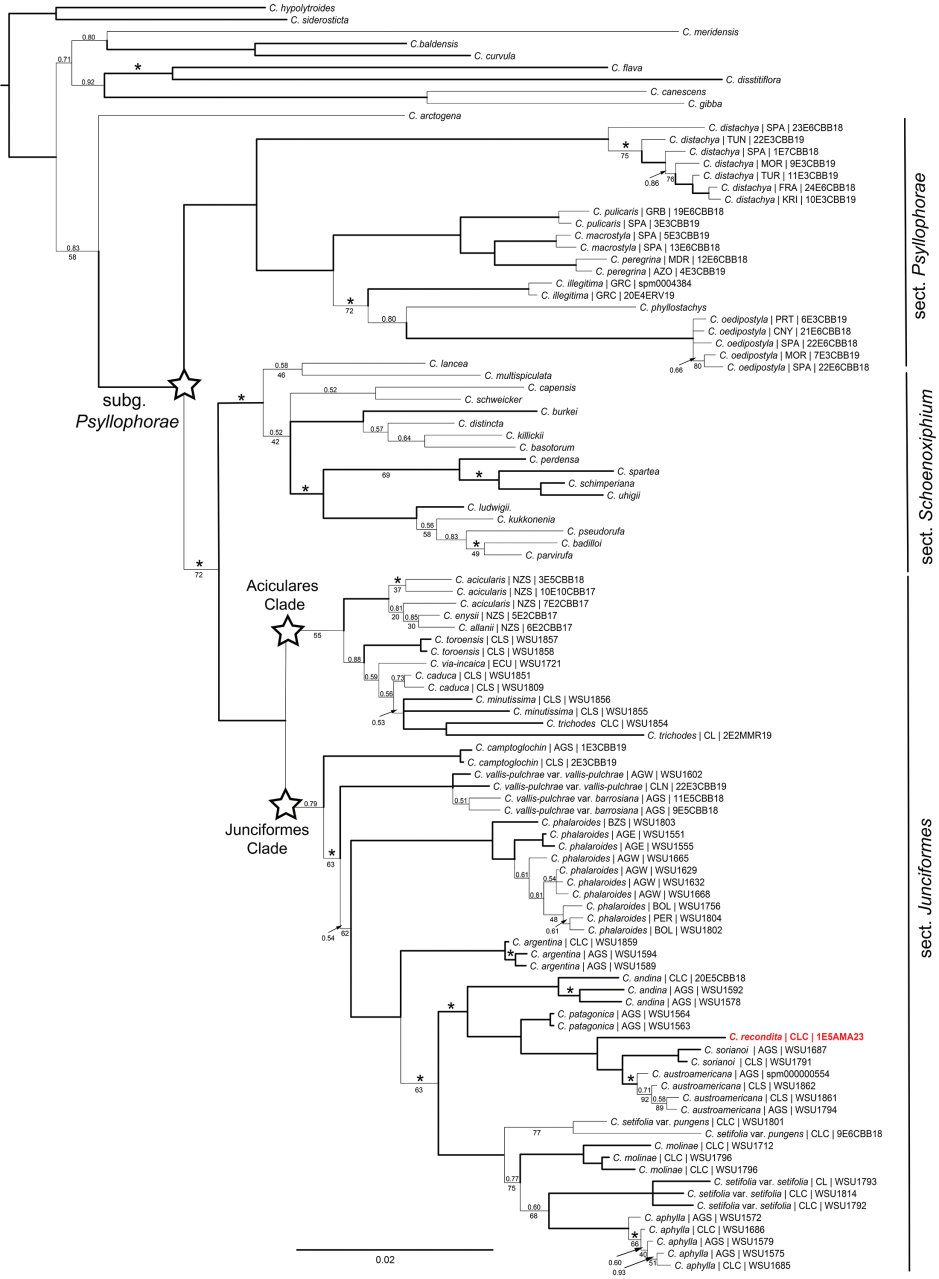
Phylogram resulting from BI analysis using MrBayes of the multiaccession matrix for Carexsubg.Psyllophorae. Branch support is not indicated when PP=1.00/BS = 100. Posterior probability (PP) support is given above branches while MLBS support is given below branches. Asterisks above branches indicate BI 100 > PP ≥ 0.95. Bold thick branches indicate nodes supported by MLBS ≥ 80 and/or BIPP ≥ 0.95. ML support BS ≤ 80 is given below branches. The newly sequenced voucher of *C.recondita* is displayed in bold red letters. Tip labels include the geographical origin of the specimen using TDWG level 3 regions abbreviations (“botanical countries”; Brummitt 2001).

**Figure 4.**
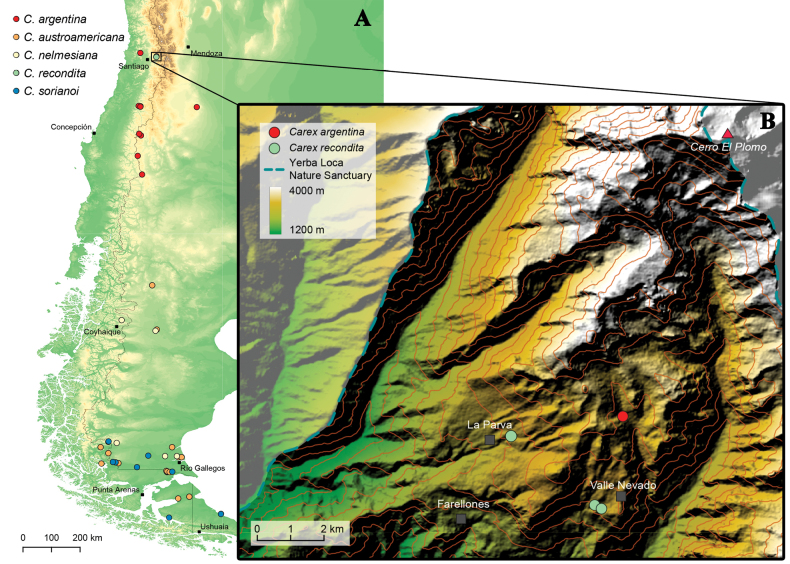
Occurrence map of Carexsect.Junciformes species cited in this work **A** occurrence map of five Patagonian species of Carexsect.Junciformes. Species locations are depicted in colored circles and some cities are represented as black squares **B** detailed map of the area of occurrence of *C.recondita* (light-green circles). Another species occurring in the area, *C.argentina*, is depicted by red circles. The highlighted region corresponds to the Yerba Loca Nature Sanctuary. Altitude was represented by a color palette and contour lines, representing elevation every 200 meters. For orientation purposes, some landmarks were depicted on the map.

#### Vernacular name.

We propose the common name for this species to be “Carex de las nieves”.

#### Conservation status.

*C.recondita* has an area of occupancy (AOO) smaller than 10 km^2^, and only a single population (three subsets) of approximately < 200 individuals has been reported. Although the entire known individuals of this species are located in protected land (Yerba Loca Nature Sanctuary), the management of the area that is effectively being protected only includes the watershed of the La Yerba Loca stream, which is located 4 km west (in straight line) and approximately 1000 m below the area in which *C.recondita* grows. Notwithstanding this, the new species grows within one of the most crowded ski circuits of Chile (La Parva and Farellones-Valle Nevado circuit), on an area contiguous to one of its main roads, which has also been affected by exotic plant invasions (Cavieres et al. 2008). Road construction and maintenance works seem like a plausible threat to the future conservation of the population. The scarcity of observed individuals and the lack of previous reports or collections for this area suggests that *C.recondita* might be extremely rare and thus vulnerable to disturbances. The real extent of its natural distribution is unknown, and its area of occurrence might be greater than estimated by current data. However, and according to our data and observations, we propose that this species is better assessed as ‘Critically Endangered’ (CR; B2ab (ii-iv) + C2a (i)), according to the IUCN Red List criteria (IUCN 2017). Further searching of this species will contribute to expanding the knowledge about its presence and might help proposing a more robust assessment.

#### Notes.

*C.recondita* is a tuft-forming species with stiff leaves and an acaulescent spike burrowed between the leaves, a type of growth form shared with many other Patagonian species of sect. Junciformes and recurrent in South American *Carex* species (see Jiménez-Mejías et al. 2021b). This type of growth might be an adaptation to harsh environments such as the high Andes or the Patagonian steppe (Körner 2021, pp. 37–38). This made *C.recondita* remain unnoticed until now, despite growing just beside a busy road in a popular skiing holiday area barely over one hour by road from Santiago, the most populated city of Chile. Some of the studied material included inflorescences with involucral bracts bearing -apparently- infertile female flowers.

#### Additional specimens examined (*paratypes*).

Chile. Región Metropolitana de Santiago: Provincia de Santiago, camino entre Farellones y Valle Nevado, laderas por debajo de la curva 14, piso altoandino, 2853 m a.s.l., 33°21.46188'S, 70°15.32352'W, 20 December 2022, *M.T.K. Arroyo, V. Robles, K. Robles, M. Acevedo & L. Retamal 29576* (CONC). Provincia de Santiago, Farellones, a 1 km de la Parva. 2674 m a.s.l., 33°20'S, 70°17'W, 1 March 2007, *M. Mihoc 777* (CONC 178536).

## Supplementary Material

XML Treatment for Carex recondita

## ﻿Additional information

### Author ORCIDs

Paulo Muñoz-Schüler https://orcid.org/0009-0001-7070-4121

Ana Morales-Alonso https://orcid.org/0000-0003-1497-1070

José Ignacio Márquez-Corro https://orcid.org/0000-0003-4277-2933

Mary T. K. Arroyo https://orcid.org/0000-0001-6315-0426

Santiago Martín-Bravo https://orcid.org/0000-0003-0626-0770

Pedro Jiménez-Mejías https://orcid.org/0000-0003-2815-4477

### Data availability

All of the data that support the findings of this study are available in the main text.
